# Bridging Andrology and Oncology: Prognostic Indicators of Cancer Among Infertile Men

**DOI:** 10.3390/cimb47110930

**Published:** 2025-11-08

**Authors:** Athanasios Zachariou, Efthalia Moustakli, Athanasios Zikopoulos, Maria Filiponi, Anastasios Potiris, Nikolaos Kathopoulis, Themos Grigoriadis, Maria Tzeli, Nikolaos Machairiotis, Ekaterini Domali, Nikolaos Thomakos, Sofoklis Stavros

**Affiliations:** 1 Department of Urology, School of Medicine, University of Ioannina, 45110 Ioannina, Greece; zahariou@otenet.gr; 2 Department of Nursing, School of Health Sciences, University of Ioannina, 4th Kilometer National Highway Str. Ioannina-Athens, 45500 Ioannina, Greece; 3 Torbay and South Devon NHS Foundation Trust, Lowes Brg, Torquay TQ2 7AA, UK; thanzik92@gmail.com; 4 Urology Outpatient Department, KENTAVROS Rehabilitation Centre, 38222 Volos, Greece; mfilipou@gmail.com; 5 Third Department of Obstetrics and Gynecology, University General Hospital “ATTIKON”, Medical School, National and Kapodistrian University of Athens, 12462 Athens, Greece; apotiris@med.uoa.gr (A.P.); nikolaosmachairiotis@gmail.com (N.M.); sfstavrou@med.uoa.gr (S.S.); 6 First Department of Obstetrics and Gynecology, Alexandra Hospital, Medical School, National and Kapodistrian University of Athens, 11528 Athens, Greece; nickatho@gmail.com (N.K.); tgregos@med.uoa.gr (T.G.); kdomali@yahoo.fr (E.D.); thomakir@hotmail.com (N.T.); 7 Department of Midwifery, Faculty of Health and Caring Sciences, University of West Attica, 12243 Athens, Greece; mtzeli@uniwa.gr

**Keywords:** male infertility, spermatogenesis impairment, cancer risk, BRCA mutations, mismatch repair genes, epigenetic dysregulation, metabolomics, precision medicine

## Abstract

Approximately 7% of males globally suffer from male infertility, which is becoming more widely acknowledged as a clinical indicator of potential health hazards as well as a cause of reproductive failure. Among these, cancer has become a significant worry due to mounting evidence that spermatogenesis impairment is associated with increased risk of prostate, testicular, and other cancers. Male infertility may be an early clinical manifestation of systemic genomic instability due to shared biological pathways, such as Y-chromosome microdeletions (AZF regions), germline DNA repair defects, mutations in tumor suppressor genes (e.g., *BRCA1/2*, *TP53*), mismatch repair gene mutations (e.g., *MLH1*, *MSH2*), and dysregulated epigenetic profiles. This narrative review covers the most recent research on prognostic markers of cancer in infertile men. These include molecular biomarkers such as genetic, epigenetic, and proteomic signatures; endocrine and hormonal profiles; and clinical predictors such as azoospermia, severe oligozoospermia, and a history of cryptorchidism. The possibility of incorporating these indicators into risk stratification models for precision medicine and early cancer surveillance is highlighted. For this high-risk group, bridging the domains of andrology and oncology may allow for better counseling, earlier detection, and focused therapies.

## 1. Introduction

Approximately 40–50% of infertility cases are due to male factors, making it a global health concern that affects 15% of couples of reproductive age. While male infertility has traditionally been regarded as a reproductive disorder, emerging evidence now identifies it as an early marker of systemic disease, particularly malignancy. This review uniquely bridges the fields of andrology and oncology by synthesizing molecular, hormonal, and clinical predictors of cancer risk among infertile men, aiming to highlight translational opportunities for precision-based early cancer surveillance.

Male infertility is a complex condition with congenital, genetic, endocrine, environmental, and idiopathic determinants [1]. Although impaired male fertility has long been viewed primarily as a reproductive health problem, there is growing recognition that it can also serve as a clinical warning sign of systemic disease, associated with higher morbidity and mortality. Among these systemic risks, cancer represents one of the most significant and clinically relevant correlations [2].

According to epidemiological research, males who are infertile, especially those who have azoospermia or severe oligozoospermia, are more likely to develop cancer than their fertile counterparts [3]. Reports from large cohort studies have indicated a two- to three-fold increased relative risk, particularly for testicular germ cell tumors (the strongest association), hematological malignancies, and to a lesser extent, prostate cancer [4]. Common genetic and molecular pathways lend biological plausibility to this association: chromosomal abnormalities like Y-chromosome microdeletions in AZF regions, tumor suppressor gene mutations (e.g., *TP53*, *RB1*), and germline defects in DNA repair (e.g., *MLH1*, *MSH2*, *MSH6*, *BRCA1/2*) have all been linked to both impaired spermatogenesis and carcinogenesis [5]. Furthermore, systemic genomic instability may be reflected in altered sperm epigenetic profiles, such as aberrant DNA methylation and imprinting mistakes, which could predispose affected men to cancer in later life [6,7].

Infertility may offer a special window of opportunity for early cancer risk detection from a clinical standpoint [8,9]. In many andrology clinics, the diagnostic workup already includes genetic testing, hormone profile, and semen analysis. Implementing customized surveillance systems and risk categorization may be made possible by combining this data with newly discovered biomarkers [10]. Nevertheless, there is now no standardized method for screening for cancer in infertile males, and there is still little use of research findings in clinical settings, despite mounting evidence [11,12].

This narrative review aims to synthesize the existing data on prognostic markers of cancer in infertile men to bridge the domains of andrology and oncology. In addition to summarizing clinical, hormonal, and genetic biomarkers with possible prognostic significance, we also examine epidemiological data and emphasize common pathophysiological pathways. To enhance early cancer identification and improve clinical outcomes in this at-risk population, we conclude by discussing the implications for risk stratification, screening techniques, and future research prospects.

## 2. Methods

This study was intended to synthesize the most recent data on predictive markers of cancer in infertile males. It was structured as a narrative review. The following keyword combinations were used in a thorough literature search up to August 2025 in PubMed/MEDLINE, Scopus, and Web of Science: male infertility, azoospermia, oligozoospermia, cancer risk, testicular cancer, prostate cancer, hematologic malignancies, genetic biomarkers, epigenetics, and DNA repair. Additional research was found by screening the reference lists of pertinent papers.

Preclinical and clinical research, such as genetic association studies, case–control studies, epidemiological cohorts, and mechanistic studies, were taken into account. Systematic reviews, meta-analyses, large population-based research, and high-quality observational cohorts were prioritized. Single case reports, conference abstracts without full text, and articles not published in English were not included.

An official risk-of-bias assessment and meta-analysis were not conducted due to the narrative design. To emphasize important epidemiological correlations, common biological pathways, and new prognostic indicators, the findings were instead critically evaluated and compiled. Evidence with translational significance to clinical practice and cancer risk assessment was given special attention.

## 3. Male Infertility and Cancer Risk: The Evidence

Over the past 20 years, several extensive population-based studies have provided evidence supporting the link between male infertility and an elevated risk of cancer [13,14]. Scandinavian registries provided the first evidence that males with low-quality semen had a markedly increased risk of testicular germ cell tumors (TGCTs) in comparison to fertile controls. The range of cancers linked to infertility has been broadened by more recent studies, and now includes hematologic, colorectal, prostate, and melanoma cancers [12,15].

### 3.1. Testicular Cancer

The cancer that is most frequently associated with infertility is testicular germ cell tumor [15]. Over 30,000 men were assessed for infertility as part of a historic Danish cohort study, and among those with aberrant semen characteristics, the standardized incidence ratio (SIR) for testicular cancer was 1.6 (95% CI: 1.3–1.9). In comparison to the general population, the risk was almost three times higher for azoospermic men [16]. Given that germ cell cancers and spermatogenesis disorders share embryonic origins and may both result from testicular dysgenesis syndrome (TDS), which includes cryptorchidism, hypospadias, and decreased Leydig/Sertoli cell activity, this substantial relationship makes biological sense [17,18].

### 3.2. Prostate Cancer

Studies on the risk of prostate cancer in infertile males have produced conflicting results; some have found a higher risk, while others have suggested a decreased risk because of less exposure to androgen [19]. A twofold higher incidence of high-grade prostate cancer (Gleason score ≥8) but not of low-grade illness was found in a large US study involving over 76,000 infertile men. Even though later research has yielded contradictory results, it is still possible that infertility might be a sign of aggressive prostate cancer biology because oncogenesis and spermatogenic failure are both caused by common DNA repair deficits (such as BRCA2 mutations) [20].

Research on the risk of prostate cancer in guys who are infertile has yielded mixed findings; some indicate a higher risk, while others suggest a lower risk, maybe as a result of a decreased lifetime exposure to androgen. In contrast to Wirén et al. (2013) and Laukhtina et al. (2021), who found either no association or a slightly lower risk in population-based cohorts, Walsh et al. (2010) discovered a twofold greater incidence of high-grade prostate cancer among infertile males. Differences in cohort design, definitions of infertility, and confounding by age or hormonal milieu could all contribute to this discrepancy [19,20,21].

One significant moderator of this association is advanced age. Infertility is rarely a direct result of prostate pathology in this situation because infertility is normally identified decades earlier, whereas prostate cancer usually starts in the sixth decade of life [21]. Instead, OS, metabolic syndrome, and cumulative DNA damage are examples of age-related comorbidities that may affect spermatogenesis on their own and then contribute to prostate carcinogenesis. Therefore, infertility might be an early indicator of systemic genomic instability that occurs before age-related prostate cancer rather than arising as its consequence [22].

### 3.3. Hematologic and Other Malignancies

Men with poor semen quality had higher rates of hematologic malignancies, such as multiple myeloma, acute myeloid leukemia, and non-Hodgkin lymphoma, according to several registry-based studies [13,23]. Furthermore, albeit with smaller effect sizes and less consistent evidence, elevated risks have been noted for bladder cancer, thyroid cancer, colorectal cancer, and melanoma [24,25]. Together, these results imply that male infertility may not only be related to urogenital cancers but also represent a systemic propensity to cancer. Table 1 summarizes the major findings of important epidemiological research that link male infertility to cancer risk [11].

### 3.4. Mortality and Longitudinal Outcomes

According to reports, infertile males had a lower life expectancy and a higher all-cause mortality rate than their fertile counterparts, in addition to incident cancer diagnoses [35]. Those with azoospermia experience the greatest burden, showing a ~2.2-fold higher risk of premature death, largely from malignancy [36]. These findings underscore the need to recognize infertility not merely as a reproductive limitation but as an early clinical signal of systemic disease, warranting long-term oncologic and metabolic surveillance.

## 4. Shared Biological and Pathological Mechanisms

Rather than a straightforward cause-and-effect relationship, the observed correlation between male infertility and an increased risk of cancer is probably caused by overlapping biochemical pathways [14]. Hormonal imbalances, environmental exposures, epigenetic dysregulation, and genetic predisposition are some of the explanations that have been suggested. A state of genomic instability might be produced by these variables coming together, which would hinder spermatogenesis and increase the risk of cancer [37,38].

### 4.1. Genetic Susceptibility

For male infertility and cancer risk, one of the most compelling molecular explanations is genetic predisposition. Particularly significant are germline abnormalities in DNA repair mechanisms, which jeopardize genomic stability in somatic tissues as well as germ cells [39,40]. Lynch syndrome is caused by mutations in mismatch repair (MMR) genes, which include *MLH1*, *MSH2*, *MSH6*, and *PMS2*. These mutations have been linked to impaired spermatogenesis and may concurrently put afflicted men at risk for colorectal, endometrial, and urogenital cancers [41,42]. Similarly, non-obstructive azoospermia has been reported in some men carrying mutations in homologous recombination repair genes, particularly BRCA2, which also confer elevated risk of aggressive prostate, pancreatic, ovarian, and breast malignancies [43,44]. The idea that male infertility may be a clinical sign of hereditary cancer susceptibility has been supported by the discovery of rare germline mutations in additional DNA damage response genes, such as *CHEK2* and *ATM*, in males with defective spermatogenesis [26].

Both spermatogenesis and oncogenesis depend heavily on tumor suppressor genes, cell-cycle regulators, and DNA repair genes [45,46,47]. Malignant transformation is more likely when TP53 or RB1 loss-of-function mutations affect spermatogonial maturation and interfere with the death of damaged germ cells [48]. This shared vulnerability is further demonstrated by chromosomal abnormalities: men with Klinefelter syndrome (47, XXY) have severe spermatogenic failure and are significantly more likely to develop breast cancer, mediastinal germ cell tumors, and several hematologic malignancies [49,50]. In addition to being known causes of male infertility, Y-chromosome microdeletions, specifically in the AZFa, AZFb, and AZFc areas, may potentially increase the risk of cancer by disrupting genes that regulate cell division and apoptosis, although supporting evidence remains limited [51,52].

### 4.2. Epigenetic Dysregulation

Male infertility and cancer risk are linked by a crucial mechanism that goes beyond genetic changes: epigenetic dysregulation. Histone modification, DNA methylation, and short non-coding RNA regulation are all examples of the exact epigenetic programming necessary for proper spermatogenesis [53,54]. Infertile men have been found to exhibit aberrant methylation patterns at imprinted loci, including H19 and MEST, which may be indicative of broader genomic instability that predisposes to cancer [55]. Global hypomethylation, which is frequently observed in sperm from men with severe azoospermia or oligozoospermia, can result in loss of imprinting and oncogene activation, and is also present in cancer cells [56,57].

Furthermore, chromatin compaction in sperm is compromised by incorrect histone retention and altered protamine-to-histone ratios, which may impact genome integrity and increase vulnerability to DNA damage [58,59]. Genomic instability and an ideal environment for carcinogenesis may be further exacerbated by dysregulated short non-coding RNAs, such as piwi-interacting RNAs (piRNAs), which typically silence transposable elements in germ cells. When taken as a whole, these epigenetic abnormalities suggest that compromised spermatogenesis could serve as a proxy for systemic cellular instability and increased cancer risk [60,61].

### 4.3. Hormonal Imbalance, Infertility, and Systemic Disease

Male infertility and the risk of developing cancer are both influenced by hormonal abnormalities. Feedback disruption causes increased gonadotropins (FSH and LH) in men with primary testicular failure, which is indicative of continuous damage to germ cells [62,63]. The testicular microenvironment could be altered due to chronic hormonal dysregulation, increasing oxidative stress (OS) and impairing DNA repair. In addition to infertility, aggressive types of prostate cancer, and metabolic abnormalities that may increase the risk of developing cancer, low serum testosterone—a typical finding in hypogonadal men—has also been connected to these conditions [20,64]. Hyperestrogenism, however, may promote aberrant cell proliferation and increase the risk of cancer in conditions like Klinefelter syndrome. Endocrine function, reproductive health, and systemic disease are intricately intertwined, with hormonal imbalance serving both as a consequence of impaired spermatogenesis and a possible facilitator of cancer development [65,66].

### 4.4. Environmental and Lifestyle Exposure

The association between cancer risk and male infertility is further influenced by environmental and lifestyle factors. Men who are infertile frequently encounter oxidative stressors, such as environmental pollutants, substances that disrupt hormones (phthalates, bisphenol A, pesticides), and occupational hazards, including ionizing radiation or heavy metals [67,68]. Infertility and carcinogenesis have the same route as OS damages sperm DNA and can encourage mutagenesis in somatic tissues. Obesity, smoking, alcohol consumption, and sedentary activity are examples of lifestyle factors that exacerbate oxidative damage, disrupt hormonal balance, and raise systemic inflammation [69,70]. These factors all contribute to poor spermatogenesis and an increased risk of cancer. When combined with genetic and epigenetic vulnerabilities, these exposures may create a “perfect storm” in which infertility represents a marker of broader susceptibility to cancer. The shared mechanisms linking infertility and cancer are summarized in Table 1.

### 4.5. Biological Pathways Linking Inferility and Genomic Stability

Male infertility and systemic genomic instability are related in several mechanisms that control germ cell development, cell survival, and DNA integrity. Specifically in homologous recombination and mismatch-repair systems, faults in the DNA damage-response network are among the most important processes. Mutations that affect spermatogenesis and predispose to cancer are caused by germline changes in genes such as *BRCA1*, *BRCA2*, *ATM*, *MLH1*, and *MSH2*, which compromise the repair of DNA double-strand breaks [71,72,73].

Mutations in *TP53*, *RB1*, or *CDKN2A* cause cell-cycle checkpoint dysregulation, which upsets the equilibrium between germ-cell proliferation and death. Malignant transformation is facilitated when these checkpoints are missed, allowing genetically faulty spermatogonia and somatic cells to survive [74].

Additionally, OS signaling plays a role in carcinogenesis and infertility. The testicular microenvironment’s overproduction of ROS damages sperm DNA and triggers mutagenesis cascades that are mediated by the NF-κB and MAPK pathways. Therefore, chronic OS causes genomic instability in somatic tissues in addition to lowering sperm quality [75,76].

Epigenetic reprogramming adds another level of interconnection. Global DNA hypomethylation, loss of imprinting, and abnormal chromatin compaction are caused by the dysregulation of short non-coding RNAs such as piRNAs and the aberrant activity of DNA-methyltransferases (DNMTs) and histone-modifying enzymes. Both poor spermatogenesis and cancer development are characterized by these epigenetic abnormalities [77].

**Figure 1:**
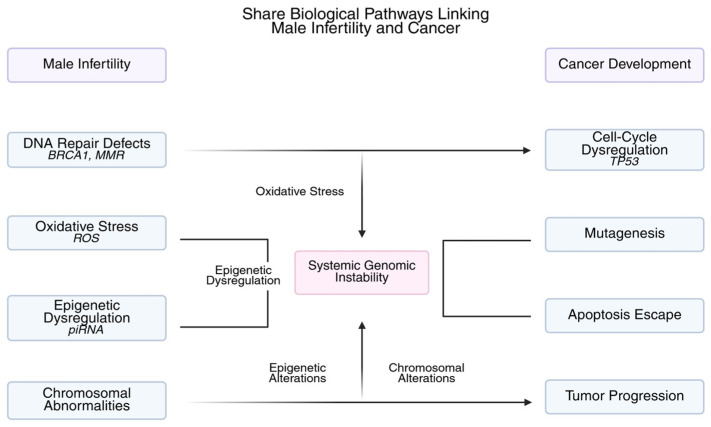
**Shared Biological Pathways Linking Male Infertility and Cancer.** Defects in DNA repair (*BRCA1*, *MMR*), oxidative stress (ROS), and epigenetic dysregulation (*piRNA*) contribute to systemic genomic instability, linking impaired spermatogenesis with tumor development. These shared mechanisms underlie cell-cycle dysregulation, mutagenesis, and tumor progression.

The combination of these overlapping oxidative, epigenetic, and genetic changes results in a biological continuum, where systemic genomic instability manifests clinically as decreased spermatogenesis. Comprehending these common pathways offers a mechanistic basis for incorporating molecular indicators into the evaluation of cancer risk in infertile males [78,79]. These interrelated mechanisms are illustrated in Figure 1.

## 5. Prognostic Indicators of Cancer Risk in Infertile Men

A thorough strategy that incorporates clinical traits, hormone profiles, semen measurements, and molecular indicators is needed to identify infertile men who are at higher risk for cancer. In cancer and andrology clinics, prognostic indications can direct counseling, early surveillance, and risk classification [80,81].

### 5.1. Clinical Indicators

Among infertile men, there is a clear correlation between certain clinical characteristics and an increased risk of cancer. The risk of testicular and hematologic cancers is two to three times higher in individuals with significant spermatogenic abnormalities, especially azoospermia and severe oligozoospermia [14]. A history of testicular dysgenesis syndrome, hypospadias, or cryptorchidism increases risk further since these conditions represent developmental abnormalities that predispose to poor spermatogenesis and malignancy. Comorbid conditions like metabolic syndrome and advanced age upon infertility diagnosis may also have prognostic significance [82,83].

### 5.2. Hormonal Profiles

Indirect indicators of underlying systemic or testicular pathology may be found in endocrine parameters. Reduced spermatogenesis is associated with elevated FSH and LH levels, which are suggestive of early testicular failure. They may also indicate ongoing death of germ cells and genomic stress. While aberrant cell proliferation may be a result of altered estrogen/testosterone ratios, low blood testosterone has been variably associated with aggressive prostate cancer phenotypes as well as infertility. These hormone measurements can improve risk prediction models when paired with clinical data [84,85].

### 5.3. Semen Parameters

Semen analysis, which offers predictive information beyond reproductive capacity, is still the mainstay of evaluating male infertility. Cancer risk has been linked to sperm concentration, motility, and morphology; the most severe abnormalities (azoospermia, severe oligozoospermia) are associated with the highest risk. Oxidative damage and increased sperm DNA fragmentation may also serve as early molecular markers of genomic instability that coincide with cancer susceptibility [86,87].

### 5.4. Genetic Biomarkers

Men with an inherited propensity for cancer can be identified by genetic screening. In addition to impairing spermatogenesis, mutations in the *BRCA2*, *CHEK2*, *ATM*, *TP53*, *RB1*, and *MMR* genes (*MLH1*, *MSH2*, *MSH6*, *PMS2*) significantly increase the risk of developing cancer [88,89]. Chromosome abnormalities such as Y-chromosome AZF microdeletions and Klinefelter syndrome (47,XXY) are strongly linked to infertility and specific cancers. For males who have a family history of cancer or severe, idiopathic infertility, genetic assessment may be especially helpful [90,91].

### 5.5. Epigenetic and Molecular Biomarkers

Systemic genomic instability is also reflected in epigenetic markers such as dysregulated piRNAs, altered histone-to-protamine ratios, and aberrant DNA methylation at imprinted loci (*H19*, *MEST*) [92,93]. Although they are still mostly in the research stage, proteomic and metabolomic patterns in seminal plasma are being investigated as possible indicators of early cancer risk. Precision risk stratification may become possible in the future if these molecular data are integrated with clinical and genetic indications [94,95,96].

### 5.6. Toward a Risk Stratification Model

A multi-parametric risk score that incorporates clinical, hormonal, genetic, and molecular markers could be a useful method for detecting high-risk infertile males [97]. Men who have pathogenic germline mutations, aberrant hormonal profiles, or severe spermatogenic abnormalities may be given preference for specialized surveillance programs that include hematologic examination, PSA monitoring, or testicular imaging. A paradigm like this would allow for early intervention, bridge the gap between oncology and andrology, and enhance long-term results [98,99]. Collectively, these parameters can be integrated into multiparametric models to improve cancer risk prediction among infertile men (Table 2).

## 6. Translational and Clinical Implications

### 6.1. Predictive Biomarkers in Male Infertility

Early adulthood is a common time for male infertility examinations, which is a useful chance to identify those who are more likely to develop cancer before symptoms appear [121]. Semen analysis, hormonal profiling, and, in certain situations, genetic testing is already part of the diagnostic process for infertile males. These studies offer predictive data pertinent to systemic health in addition to assisting with reproductive counseling. Therefore, routine reproductive examinations could become more comprehensive health surveillance platforms if cancer risk assessment is included into infertility clinics [122,123]. In particular, high-risk populations of men who have pathogenic germline mutations, aberrant hormonal profiles, or severe spermatogenic abnormalities may need early screening. For instance, men with *BRCA2* or mismatch repair gene mutations may need more thorough screening for prostate and colorectal cancer, whereas azoospermic males with increased gonadotropins or Y-chromosome microdeletions may benefit from testicular imaging and routine surveillance.

### 6.2. Counseling and Ethical Considerations

Important counseling and ethical issues are brought up when cancer susceptibility is discovered during an infertility evaluation [124]. Conversations on hereditary cancer syndromes or long-term cancer risk may come as a surprise to patients looking for fertility solutions. Thus, clinicians need to give patients balanced information, outlining the significance of aberrant findings for both reproduction and cancer [125]. Careful pre-test counseling is necessary for genetic testing, in particular, to address the potential for incidental discoveries, psychosocial discomfort, and family member consequences [126]. The advantages of early detection should be emphasized in informed consent, but so should the drawbacks of predictive testing and the possibility of error in risk assessments. To ensure that males and their partners can make informed choices without excessive worry or stigmatization, it is crucial to establish proper communication mechanisms [127].

### 6.3. Surveillance and Early Detection Strategies

The translation of prognostic markers into practice requires structured surveillance algorithms [128]. Men harboring *BRCA2* or *MMR* mutations may benefit from earlier prostate-specific antigen (PSA) testing or colonoscopic screening, whereas azoospermic men with Y-chromosome deletions could undergo periodic testicular ultrasonography and serum tumor-marker assessment. Integrating these evaluations within fertility clinics provides a practical platform for early cancer detection before symptomatic onset.

Emerging liquid-biopsy and multi-omics assays of seminal plasma may further refine such precision-based surveillance. Prostate-specific antigen monitoring for men with pathogenic *BRCA2* variants, testicular ultrasound and tumor marker evaluation for men at high risk of testicular cancer, and hematological evaluations for those with severe spermatogenic failure and inexplicable systemic symptoms are a few examples [129].

To prevent overdiagnosis and unnecessary interventions, surveillance must be tailored to each individual’s risk profile. Personalized and cost-effective programs are possible using precision medicine techniques that integrate clinical, hormonal, and molecular indications into risk classification models [130,131]. Finally, the need for a thorough long-term health-management plan that goes beyond cancer surveillance has been highlighted by the growing correlations between infertility and cardiometabolic disease and premature mortality [132,133].

### 6.4. Multidisciplinary Collaboration and Health System Needs

Coordinated care across disciplines is necessary for the successful implementation of cancer risk assessment among infertile men. Though they are in a unique position to recognize men who are at risk, andrologists frequently need to refer patients to primary care physicians, genetic counselors, and oncologists for appropriate management [134,135]. The creation of integrated care pathways can guarantee that prognostic markers found in the fertility clinic are converted into useful monitoring and prevention plans. To change the perspective of infertility from a solely reproductive problem to a sign of systemic health vulnerability, awareness campaigns and professional education are required at the health system level [136,137]. The development of evidence-based screening guidelines and the improvement of risk models will also require investments in registries and data-sharing programs. Ultimately, improving reproductive outcomes and promoting cancer prevention and early diagnosis in a high-risk but underappreciated group are two benefits of bridging the andrology-oncology divide [138,139].

## 7. Future Directions and Research Gaps

Although substantial progress has been made in elucidating the connection between male infertility and cancer, significant gaps remain in both mechanistic understanding and clinical translation. To go from association to causality and from prediction to prevention, these gaps must be filled [80,140].

### 7.1. Future Directions and Research Gaps

There is still little prospective evidence linking male infertility to cancer, although several registry and retrospective studies indicate this link. The complexity of spermatogenic dysfunction and its evolution over time may not be adequately captured by the majority of existing data, which rely on semen characteristics at a single time point [14,141]. There is an urgent need for longitudinal cohort studies that include regular semen testing, uniform definitions of infertility, and long-term follow-up for cancer outcomes. Such investigations would shed light on whether infertility is a reflection of underlying genetic predispositions or if it predicts cancer on its own [14,142]. Identifying temporal trends, such as whether certain cancers develop preferentially during particular time windows after an infertility diagnosis, would also be made possible by them [143].

### 7.2. Multi-Omics and Biomarker Discovery

Advances in genomics, transcriptomics, epigenomics, proteomics, and metabolomics provide unprecedented opportunities to unravel the biological links between impaired spermatogenesis and oncogenesis [144,145]. Novel biomarkers that not only reveal common biological pathways but also more precisely stratify cancer risk than clinical signs alone may be found using multi-omics techniques [146,147]. Proteomic profiles of seminal plasma or epigenomic signals in sperm DNA, for instance, could be early markers of systemic genomic instability. Before these potential biomarkers are used in clinical settings and across a range of demographics, they must first be thoroughly validated. One of the main challenges for the upcoming ten years of study will be to ensure clinical utility and reproducibility [148,149].

### 7.3. Emerging Technologies for Surveillance

Early cancer detection in infertile men may be possible thanks to advancements in non-invasive diagnostics. Techniques for liquid biopsies, such as the detection of cell-free DNA (cfDNA) and circulating tumor DNA (ctDNA), may offer sensitive indicators of occult cancer [150,151]. Similarly, RNA profiles and extracellular vesicles produced from sperm may be readily available biomarkers of systemic illness. These technologies are still in the experimental stage, but they have the potential to lessen dependence on invasive procedures and supplement traditional surveillance approaches. To evaluate patient acceptance, cost-effectiveness, and feasibility in relation to cancer risk associated with infertility, early pilot studies are required [152,153].

### 7.4. Artificial Intelligence and Predictive Models

Clinicians have difficulties due to the intricacy of combining clinical, hormonal, genetic, and molecular data. By making it possible to create multivariate prediction models, artificial intelligence and machine learning techniques present viable remedies [154,155]. These tools could create individualized surveillance suggestions and categorize infertile men into risk groups. Large infertility registries, for example, may teach machines to spot patterns that traditional statistical techniques would miss. However, to guarantee fair and clinically significant results, the use of AI in this field would necessitate strong datasets, close attention to algorithmic openness, and anti-bias measures [156].

### 7.5. Clinical Guidelines and Ethical Frameworks

There are currently no established standards for cancer screening in infertile males, despite mounting evidence. To create evidence-based recommendations, professional associations in cancer, urology, and andrology will need to work together internationally [21]. Guidelines must cover the psychological and ethical ramifications of predictive testing in addition to who should be examined and how frequently. Ethical frameworks will be crucial for handling incidental findings, protecting privacy, and facilitating well-informed decision-making as genomic technologies are progressively incorporated into fertility treatment [157]. The possible advantages of early detection must ultimately be weighed against the dangers of overdiagnosis and patient distress in practical application [158].

## 8. Conclusions

There is strong evidence that male infertility is a systemic health sign and that it is associated with an increased risk of cancer. Both infertility and carcinogenesis are caused by similar biological processes, such as chromosomal abnormalities, epigenetic instability, and problems in DNA repair. Promising methods for identifying males who are most at risk include clinical, hormonal, and molecular indications.

Utilizing these findings for the benefit of patients requires a bridge between oncology and andrology. Clinicians can advance earlier cancer identification, better counseling, and focused therapies by incorporating prognostic indications into risk stratification and monitoring plans. Therefore, infertility presents a crucial window of opportunity to improve long-term health outcomes for this susceptible population and advance precision medicine.

Despite mounting evidence, a number of obstacles lie in the way of practical translation, including a lack of standardized biomarkers, a small number of prospective cohorts, and ethical issues with predictive screening. Future developments will involve the creation of worldwide guidelines, AI-driven multi-omics integration, and customized cancer surveillance in infertility clinics bridging oncology and andrology. These trends will transform male infertility evaluation into a gateway for proactive health surveillance.

## Figures and Tables

**Table 1:** Summary of key mechanisms linking male infertility with increased cancer risk, highlighting major findings, representative genes or pathways, and their clinical implications.

Mechanism	Key Findings	Examples of Relevant Genes/Pathways	Notes/Implications	References
Genetic Susceptibility	Infertile men are enriched for pathogenic germline variants in cancer-associated genes; defects in DNA repair predispose to malignancy	*MLH1*, *MSH2*, *MSH6*, *PMS2*, *BRCA2*, *CHEK2*, *ATM*, *TP53*, *RB1;* Klinefelter syndrome (47,XXY); Y-chromosome AZF deletions	Shared genetic etiology: a germline defect may underlie both spermatogenic failure and elevated cancer risk	[14,26,27]
Epigenetic Dysregulation	Aberrant DNA methylation, histone retention, dysregulated small non-coding RNAs	*H19*, *MEST*, *piRNAs*	Impaired spermatogenesis may reflect systemic epigenomic instability; potential biomarker for cancer risk	[28,29]
Endocrine/Hormonal	Low testosterone, elevated FSH/LH, altered estrogen	Androgen and estrogen pathways, gonadotropin signaling	Hormonal imbalance promotes OS, alters testicular microenvironment, and may favor tumorigenesis	[30,31,32]
Environmental/Lifestyle	Exposure to toxins, endocrine disruptors, radiation; obesity, smoking	OS pathways, inflammatory mediators	Interacts with genetic and epigenetic susceptibility, amplifying cancer risk; reinforces multifactorial etiology	[33,34]

**Table 2:** Prognostic indicators of cancer among infertile men.

Indicator Category	Specific Marker/Feature	Associated Cancer Types	Evidence Strength	Notes/Clinical Implications	References
Clinical	Azoospermia, severe oligozoospermia	Testicular cancer, hematologic malignancies	High	Strongest epidemiologic predictor; prioritize for surveillance	[12,36,100]
	History of cryptorchidism, hypospadias, testicular dysgenesis	Testicular cancer	Moderate–High	Reflects developmental predisposition to malignancy	[101,102]
	Advanced age at infertility evaluation	Prostate cancer, general malignancy	Moderate	Age may modify risk but less specific	[20,21]
Hormonal	Elevated FSH/LH	Testicular cancer, systemic malignancy	Moderate	Indicates primary testicular failure and germ cell stress	[103,104]
	Low serum testosterone	Aggressive prostate cancer	Moderate	Combined with clinical features improves risk stratification	[105,106]
	Altered estrogen/testosterone ratio	Testicular and prostate cancers	Low–Moderate	Potential biomarker; requires further validation	[107,108]
Semen Parameters	Sperm concentration, motility, morphology	Testicular cancer, leukemia/lymphoma	Moderate–High	Severe abnormalities (azoospermia, extreme oligozoospermia) indicate highest risk	[36,109]
	Sperm DNA fragmentation, OS	Multiple cancer types	Low–Moderate	Emerging molecular indicator of genomic instability	[110,111]
Genetic	Germline mutations in *BRCA2*, *CHEK2*, *ATM*, *TP53*, *RB1*	Prostate, breast, testicular, colorectal	High	Particularly relevant in idiopathic severe infertility or family history of cancer	[14,44,112]
	MMR gene mutations (*MLH1*, *MSH2*, *MSH6*, *PMS2)*	Colorectal, endometrial, urogenital	Moderate–High	Supports early cancer surveillance in high-risk men	[72,113]
	Klinefelter syndrome (47,XXY), Y-chromosome AZF microdeletions	Mediastinal germ cell tumors, testicular cancer	High	Strong chromosomal predictors of both infertility and malignancy	[1,114,115]
Epigenetic/Molecular	Aberrant DNA methylation (*H19*, *MEST*)	Multiple cancer types	Low–Moderate	Reflects genomic instability; investigational as biomarker	[28,116,117]
	Altered histone/protamine ratio, piRNA dysregulation	Multiple cancer types	Low	Potential molecular marker; currently research-based	[54,118]
	Seminal plasma proteomics/metabolomics	Multiple cancer types	Low	Early investigational biomarkers for risk prediction	[119,120]

## Data Availability

No new data was created or analyzed in this study. Data sharing in not applicable to this article.

## Abbreviations

The following abbreviations are used in this manuscript:

TGCTs	Testicular Germ Cell Tumors
SIR	Standardized Incidence Ratio
piRNAs	Piwi-interacting RNAs
OS	Oxidative Stress
ROS	Reactive Oxygen Species
DNMTs	DNA-methyltransferases
PSA	Prostate-specific antigen
cfDNA	Cell-free DNA
